# Editorial: Autophagy in Endocrine-Metabolic Diseases Associated With Aging

**DOI:** 10.3389/fendo.2020.00572

**Published:** 2020-08-20

**Authors:** Maria Ines Vaccaro, Vincenzo De Tata, Claudio Daniel Gonzalez

**Affiliations:** ^1^University of Buenos Aires - CONICET, Institute of Biochemistry and Molecular Medicine, Buenos Aires, Argentina; ^2^Department of Translational Research and New Technologies in Medicine and Surgery, University of Pisa, Pisa, Italy; ^3^CEMIC University Institute, Buenos Aires, Argentina

**Keywords:** autophagy, autophagy (macroautophagy), chaperon mediated autophagy (CMA), secretory autophagy, aging, diabetes mellitus, cancer, skeletal muscle atrophy

Autophagy is a highly regulated self-degradative process of cytoplasmic cellular constituents usually activated under certain conditions such as starvation or other different forms of cell stress that result in breakdown proteins and other cell components to obtain energy. Autophagy is also responsible for removing damaged or aged organelles, eliminating different pathogens and misfolded, aggregated, or altered proteins. Autophagy is an evolutionarily biologically conserved process that sequesters and delivers cytoplasmic components to the lysosome for degradation. It is also involved in the removal of cells that have undergone classical apoptosis. Autophagy is commonly associated with cell survival mechanisms: its dysregulation, however, may be also associated with cell death. In the classical view, according to the pathway that cargo follows to reach the lysosomal compartment, there are three major types of canonical degradative autophagy. These types are: microautophagy/endosomal microautophagy, chaperone mediated autophagy, and macroautophagy, the last one being characterized by the engulfment of cytoplasmic contents by a double membrane vesicle, named autophagosome. However, other non-canonical types of autophagy have been described. One of these unconventional forms is named secretory autophagy, a newly recognized process that is becoming of increasing relevance to explain the non-canonical secretion of a series of cytosolic proteins that have critical biological importance.

Disruption of autophagy is associated with aging and metabolic and degenerative diseases including cancer. This special issue contains a collection of 12 articles covering a broad range of key topics on the interplay of the different types of autophagy alterations with aging, endocrine-metabolic, and degenerative diseases.

Chaperone-mediated autophagy (CMA) represents a major mechanism for degradation of cytosolic proteins. It also plays a significant role in the regulation of lipid and carbohydrate metabolism. Dysregulation of chaperone-mediated autophagy has been found in several models of Parkinson's Disease, Alzheimer's Disease, and Huntington's Disease. Alterations in CMA may also play a role in the pathophysiology of Lateral Amyotrophic Sclerosis and other forms of degenerative disease. Alfaro et al. review in detail the potential involvement of chaperone-mediated autophagy in neurodegeneration as well as in aging, pointing out some critical gaps in knowledge as a rich substrate for further research. CMA consists of the internalization of selected cytosolic substrates into the lysosome by a mechanism that includes recognition of a pentapeptide-like KFERQ in the substrate by the chaperone hsc70, substrate presentation by the chaperone to the receptor LAMP2A, receptor multimerization, and protein internalization for degradation in the lysosome assisted by a luminal form of hsc70 (1). LAMP2A is the only known lysosomal receptor for CMA. LAMP2A localization is defective and its function impaired in cystinosis (2, 3). Zhang et al. develop and characterize human cystinotic proximal tubule cells and demonstrate that these cells are characterized by CMA defects that affect vesicular trafficking mechanisms regulating the localization in the plasma membrane of the scavenger receptor megalin.

Secretion of some proteins lacking a “signal peptide” (for instance, some cytokines, insulin-degrading enzyme, alpha-Synuclein, etc.) does not follow the canonical secretion pathway. Those proteins are secreted following different unconventional processes. One of these routes relies on autophagy; it is autophagy dependent. “Secretory autophagy” may then explain the secretion of some relevant peptides involved in several pathophysiological processes (4). Some aggregation-prone proteins, like Amyloid beta or alpha-Synuclein are secreted by secretory autophagy. Some pro-inflammatory mediators such as interleukin-1 beta also follow this non-canonical secretory process. Alteration in secretory autophagy, as extensively described by Gonzalez et al. in this special issue, may play a substantial role in degenerative and metabolic diseases and their treatment.

Skeletal muscle atrophy is a common finding in aging and many degenerative diseases. Different degrees of muscle atrophy can be achieved under diverse physiological conditions, exposure to certain drugs, or starvation. Many of the mechanisms associated with muscle atrophy remain obscure. Kretschmar et al. propose a novel role of polycystin-2, a membrane protein of the transient receptor potential family, as a regulator of skeletal muscle atrophy mediated by the modulation of mTOR in myotubes. Polycystin-2 regulates autophagy in several models, under different stimuli and in diverse tissues. However, Kretschmar et al. suggest that the polycystin-2 role in the regulation of muscle atrophy may be independent of autophagy. This article proposes new mechanisms in the field and opens spaces for further investigation in this area. Spinal and bulbar muscle atrophy is a rare disease associated with a mutation in the androgen receptor. A polyglutamine expansion in the N-terminus of the androgen receptor protein is associated with neurotoxicity. These alterations have a critical role in the pathophysiology of the disease. Cicardi et al. provide a detailed characterization of the autophagy activation and involvement in the disease initiation and progression, reveal some novel mechanisms, and suggest some potential targets for therapeutic exploration.

Hypothalamic arcuate neurons can sense the nutrient status of the organism and accordingly regulate food intake and glucose metabolism. Alterations of this neuronal network can contribute to the pathogenesis of obesity-related diseases such as type 2 diabetes mellitus (5). In their paper, Hernández-Cáceres et al. suggest that the inhibition of autophagy could be a potential mechanism for the high fat diet-induced obesity and diabetes. By using the neuronal cell line N43/5 they demonstrate that palmitic acid exposure causes inhibition of autophagy and decreased insulin sensitivity. Their results indicate that hypothalamic autophagy could represent a promising therapeutic target in diabetic patients. It has been proposed that altered autophagy can promote beta cell dysfunction in diabetic patients (6). To further clarify the relationship between autophagy and beta cell damage, Bugliani et al. explore the effects of autophagy modulation (rapamycin induction or 3-methyladenine inhibition) in human islets under conditions of ER stress. Lipotoxic (palmitate) and chemically induced (brefeldin) ER stress are associated with alteration of beta cell survival and function. Interestingly, the authors reported that, in these conditions, the promotion of autophagy by rapamycin ameliorates the function, survival, and ultrastructure of beta cells. Thus, tuning autophagy could be a useful tool for beta cell protection.

Autophagy is an important homeostatic protective mechanism, but, on the other hand, its alterations can be involved in various pathologic processes (7). The paper by Barbosa et al. reviews recent developments on the role of impaired autophagy in age-related diseases. Aging can be defined as a time–dependent deterioration of cell functioning due to damage accumulation. Autophagic activity has been shown to decrease with age, potentially contributing to the accumulation of damaged molecules and organelles. Thus, to clarify the role of dysfunctional autophagy in establishing the hallmarks of aging could help to define new anti-aging therapeutic strategies and to extend longevity.

The mTORC1 signaling pathway couples energy and nutrient abundance to the execution of cell growth, cell division and metabolism (8). Moreover, the activation of mTORC1 inhibits autophagy. The paper of Guillén and Benito reviews the role of mTORC1 in the progression of diabetes. It has been widely demonstrated that autophagy is crucial to maintain pancreatic beta cell homeostasis (9). The progression of the diabetic disease is associated with a chronic overactivation of mTORC1 and consequently with a sustained inhibition of autophagy in pancreatic beta cells. The failure of such an important protective mechanism could induce the apoptosis of pancreatic beta cells and the impairment of compensatory insulin secretion that characterize the transition to prediabetes to clinically evident type 2 diabetes.

Cancer is emerging as a complex disease where metabolic alterations and inflammatory processes play a critical role. Autophagy and autophagy related proteins are involved in cancer pathophysiology. Arroyo et al. report the cases of two patients with progressing chronic lymphocytic leukemia (CLL). They simultaneously detect autophagy protein LC3B and classical phenotypic markers used for identification of tumoral CLL B cell clones. They found that two patients with progressing CLL showed increased expression of the autophagy protein LC3B, in addition to CD38 and ZAP70 positive expression, suggesting that activation of autophagy may correlate with CLL progression even before ibrutinib treatment. Ropolo et al. present evidence of new regulatory pathways involved in autophagy induction in high resistance pancreatic tumor cells. They demonstrated that gemcitabine requires the expression of a pancreatitis associated and autophagy related protein VMP1 (10) to induce autophagy in pancreatic cancer cells PANC-1 and MIAPaCa-2 that carry activated *KRAS*. Ropolo et al. identified E2F1, a molecule regulated by the retinoblastoma pathway, as the transcription factor in gemcitabine-induced VMP1-mediated autophagy of highly resistant pancreatic cancer cells. Finally, Moutinho-Ribeiro et al. discuss the potential role of exosomes (11) (small circulating extracellular vesicles of 50–150 nm in diameter) in several aspects related to pancreatic cancer, from initiation to tumor progression and its applicability in early detection and treatment. Increasing knowledge on cancer exosomes, whose secretory mechanisms are related to autophagy, provides valuable insights on new therapeutic targets and can potentially open new strategies to treat malignant disease.

Scientific knowledge in the field of autophagy grew exponentially along the last decade. A more precise characterization of the different forms of autophagy, their molecular mechanisms and potential implications in the pathophysiology of many diseases reflect the magnitude of such advances. However, several gaps in knowledge and unmet needs remain uncovered and call for further research. Identification of molecular targets for pharmacological modulation of autophagy and its translation into clinical practice remains among the more urgent ones. The role of autophagy modulation in therapy of metabolic and degenerative diseases challenges our current knowledge and opens potentially promising avenues for future investigation.

## Author Contributions

All authors listed have made a substantial, direct and intellectual contribution to the work, and approved it for publication.

## Conflict of Interest

The authors declare that the research was conducted in the absence of any commercial or financial relationships that could be construed as a potential conflict of interest.
